# Aging alters the metabolic flux signature of the ER‐unfolded protein response in vivo in mice

**DOI:** 10.1111/acel.13558

**Published:** 2022-02-16

**Authors:** Catherine P. Ward, Lucy Peng, Samuel Yuen, John Halstead, Hector Palacios, Edna Nyangau, Hussein Mohammed, Naveed Ziari, Mohamad Dandan, Ashley E. Frakes, Holly K. Gildea, Andrew Dillin, Marc K. Hellerstein

**Affiliations:** ^1^ Department of Nutritional Sciences and Toxicology University of California Berkeley California USA; ^2^ Department of Molecular and Cellular Biology University of California Berkeley California USA

**Keywords:** aging, de novo lipogenesis, endoplasmic reticulum, proteome dynamics, proteomics, unfolded protein response

## Abstract

Age is a risk factor for numerous diseases, including neurodegenerative diseases, cancers, and diabetes. Loss of protein homeostasis is a central hallmark of aging. Activation of the endoplasmic reticulum unfolded protein response (UPR^ER^) includes changes in protein translation and membrane lipid synthesis. Using stable isotope labeling, a flux “signature” of the UPR^ER^ in vivo in mouse liver was developed by inducing ER stress with tunicamycin and measuring rates of both proteome‐wide translation and de novo lipogenesis. Several changes in protein synthesis across ontologies were noted with age, including a more dramatic suppression of translation under ER stress in aged mice as compared with young mice. Binding immunoglobulin protein (BiP) synthesis rates and mRNA levels were increased more in aged than young mice. De novo lipogenesis rates decreased under ER stress conditions in aged mice, including both triglyceride and phospholipid fractions. In young mice, a significant reduction was seen only in the triglyceride fraction. These data indicate that aged mice have an exaggerated metabolic flux response to ER stress, which may indicate that aging renders the UPR^ER^ less effective in resolving proteotoxic stress.

## Graphical abstract

Exaggerated unfolded protein response ER signature in aged animals, with higher translation of ER stress‐related proteins, lower translation of all other proteins, lower rates of de novo lipogenesis, and BiP protein synthesis and mRNA abundance higher in aged animals with tunicamycin induced ER stress.

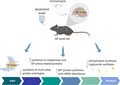

**Abbreviations**
ACUC: Animal Care and Use Committee
ATF6: activating transcription factor 6
BiP: binding immunoglobulin protein
DNL: de novo lipogenesis
ER: endoplasmic reticulum
FSR: fractional synthesis rate
GC‐MS: gas chromatography–mass spectrometry
GRP78: glucose‐regulated protein 78‐kD
IP: intraperitoneally
IRE1: inositol‐requiring enzyme‐1
LC‐MS: liquid chromatography–mass spectrometry
MIDA: mass isotopomer distribution analysis
PERK: PKR‐like ER kinase
Q‐ToF: quadrupole time of flight
RT‐qPCR: reverse transcription PCR
TLC: thin‐layer chromatography
UPR: unfolded protein response

## 1 INTRODUCTION

Loss of protein homeostasis, or proteostasis, is a central hallmark of aging and may explain why certain diseases become manifest as organisms grow older (Taylor & Dillin, 2011). Proteostasis involves coordination of the synthesis of new proteins, quality control of the proteome, and adaptive mechanisms to reduce unfolded and misfolded proteins and prevent abnormal protein aggregation (Frakes & Dillin, 2017). Proteins are synthesized in the endoplasmic reticulum (ER), and chaperones aid in the proper folding of newly synthesized proteins and assist when protein misfolding occurs. Accumulation of misfolded proteins in the ER stimulates the unfolded protein response (UPR^ER^), an integrated set of adaptations that clear misfolded protein aggregates and either restore more normal proteostasis or ultimately eliminate affected cells through apoptosis (Gardner & Walter, 2011; Walter & Ron, 2011). The UPR^ER^ consists of three downstream pathways initiated by inositol‐requiring enzyme‐1 (IRE1), PKR‐like ER kinase (PERK), and activating transcription factor 6 (ATF6), all of which are anchored in the ER membrane. ER‐localized binding immunoglobulin protein (BiP), also identified as glucose‐regulated protein 78‐kD (GRP78), is one of the responders to misfolded proteins in the ER and acts as a regulator of the UPR^ER^ (Bertolotti et al., 2000). Downstream effects include global suppression of protein translation, with the exception of key proteins involved in a rescue response such as chaperones and lipogenic proteins (Ron & Walter, 2007). If ER stress is unable to be resolved, cells undergo apoptosis (Sano & Reed, 2013). Unmitigated ER stress may be a central component of many diseases, including metabolic disorders such as fatty liver disease and insulin resistance (Hotamisligil, 2006; Wang & Kaufman, 2016).

In addition to aiding in restoration of proteostasis through slowing of global protein translation, the UPR^ER^ initiates ER membrane expansion through incorporation of fatty acids into the membrane to accommodate for aggregating proteins and chaperones that are recruited to assist in disaggregation or refolding (Schuck et al., 2009). Added ER surface may also help with the synthesis of necessary compensatory factors, such as nascent proteins and lipids. The source of these lipids incorporated into hepatocyte ER was previously unknown, but we recently discovered that the source in the liver is mobilized free fatty acids from adipose tissue during ER stress rather than their metabolic source being local de novo lipogenesis (Ward et al., 2022). Tunicamycin‐induced ER stress in mice leads to reduction of lipogenic gene expression and de novo lipogenesis in the liver (DeZwaan‐McCabe et al., 2017; Herrema et al., 2016; Ward et al., 2022). Alterations in protein and lipid fluxes, including membrane expansion, are crucial elements of the ER stress response yet remain poorly understood metabolically (Cnop et al., 2012; Fu et al., 2012; Salvadó et al., 2015; Wang & Kaufman, 2016).

Although the UPR^ER^ has been shown to decline with age in *C*. *elegans* and flies, as well as other organisms (Martínez et al., 2017), it is not fully understood how age‐induced shifts in metabolism may impair an organism's ability to handle proteotoxic stress. In *Drosophila melanogaster*, intestinal stem cells promote a regenerative response upon UPR^ER^ activation, a process which is deregulated during aging. PERK is specifically activated in intestinal stem cells; however, chronic engagement of this pathway becomes deleterious during aging (Wang et al., 2015; L. Wang et al., 2014). In *C*. *elegans*, decline of proteostasis with age was found to be reversed by expression of a constitutively active form of XBP‐1 and XBP‐1s (Taylor & Dillin, 2013). Because proteins involved in the UPR^ER^ may continue to be rapidly translated whereas translation of other proteins is suppressed through phosphorylation of eIF2α by PERK (Harding et al., 2000; Holmes et al., 2015), measurement of protein fluxes provides a potentially powerful tool for identifying UPR^ER^ regulators and signatures. In this study, we measured both proteome‐wide replacement rates and de novo lipogenesis (DNL) through in vivo stable isotope labeling. We describe a flux “signature” of the unfolded protein response in mice, which reveals proteins potentially involved in the rescue response of the UPR^ER^. Heavy water labeling in this experiment also allowed measurement of newly synthesized fatty acids, such as palmitate, and incorporation into both phospholipids and triglycerides under induced ER stress (Hellerstein et al., 1991). Phospholipids are especially of interest due to their incorporation into ER membranes under times of ER stress (van Meer et al., 2008; Volmer & Ron, 2015). We show changes in metabolic fluxes in response to ER stress that reveals less effective proteostasis with age.

## 2 RESULTS

12‐week‐old and 80‐week‐old male mice (*n* = 5 per group) were treated with 1 mg/kg tunicamycin once per day over a 4‐day treatment period or received DMSO injections (controls) to generate chronic ER stress. Tunicamycin inhibits N‐linked glycosylation, leading to the accumulation of misfolded proteins (Rutkowski et al., 2008). Mice were administered ^2^H_2_O (deuterated or heavy water), beginning at the time of the initial tunicamycin treatment (Figure S1). Proteins synthesized after tunicamycin treatment incorporate deuterium‐labeled amino acids, whereas pre‐existing proteins will not have ^2^H label in covalent C‐H bonds of amino acids, enabling the measurement of proteins that were newly synthesized during the period of exposure to tunicamycin (Holmes et al., 2015). The metabolic labeling with heavy water also quantifies newly synthesized lipids in vivo. Response to tunicamycin‐induced activation of the UPR^ER^ was characterized by proteome‐wide changes in translation rates as well as changes in de novo synthesis of palmitate incorporated into isolated phospholipid or triglyceride fractions.

### 2.1 Proteome‐wide changes in translation signatures with initiation of the UPR in young mice

**FIGURE 1:**
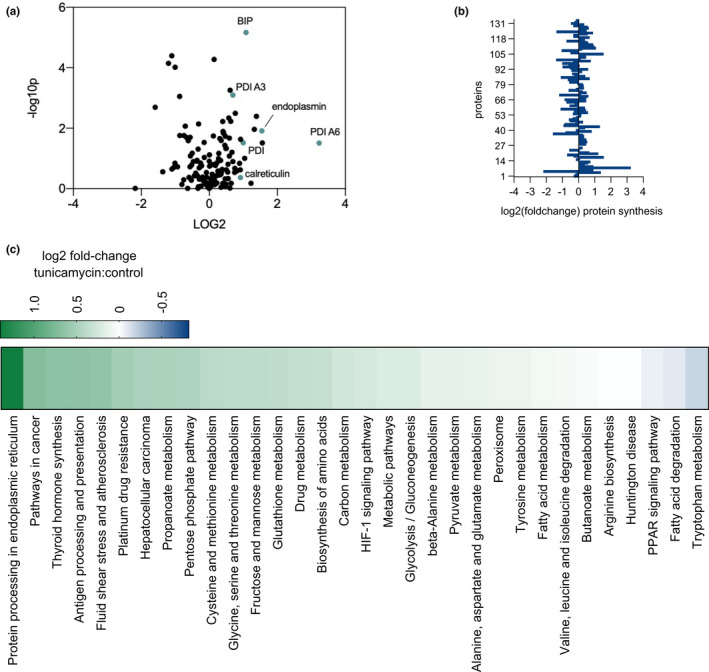
(a) Volcano plot of all hepatic proteins for which fractional synthesis rates were measured (*n* = 136) in 12‐week‐old mice over 96 h following treatment. Points expressed as log2 fold‐change tunicamycin treated/control on *x*‐axis and ‐ log10(*p* value), obtained from 2‐tailed *t*‐test (*p* ≤ 0.05), on *y*‐axis. Significantly affected ER stress proteins highlighted in blue (*p* < 0.05 as determined by 2‐tailed *t*‐test). (b) log2 fold‐change of individual protein translation rates in tunicamycin treated/control. (c) KEGG‐pathway analysis for fractional synthesis rates of proteins from 12‐week‐old mice tunicamycin treated/control. The heat map color code is shown. *n* = at least 5 proteins per pathway

The fractional synthesis or replacement rates of proteins translated during the treatment period were measured. Key UPR^ER^ proteins, including protein disulfide isomerases, BiP, endoplasmin, and calreticulin, showed greater increases in translation rates after tunicamycin relative to control values than was seen for most proteins across the proteome, during the 4‐day period post initial treatment (Figure 1a). We have elsewhere shown that global protein synthesis rates are markedly suppressed during the first 6–72 h after acute tunicamycin administration in the livers of young mice (Ward et al., 2022). Global protein translation rates here in young mice under chronic ER stress were not systematically different from controls over 4 days (Figure 1b). Proteins were organized by their KEGG‐pathways to calculate pathway specific rates of protein translation and determine the fold‐change in protein synthesis rate compared with controls, by KEGG‐pathway (Figure 1c). Under chronic ER stress conditions in young mice, protein processing in the ER was the most upregulated ontology at 2.6‐fold higher under ER stress conditions as compared with control. *Fatty acid degradation* and *PPAR signaling* were the two most suppressed ontologies. Other ontologies were mostly unaffected.

### 2.2 Age induced changes to characterized UPR signature

**FIGURE 2:**
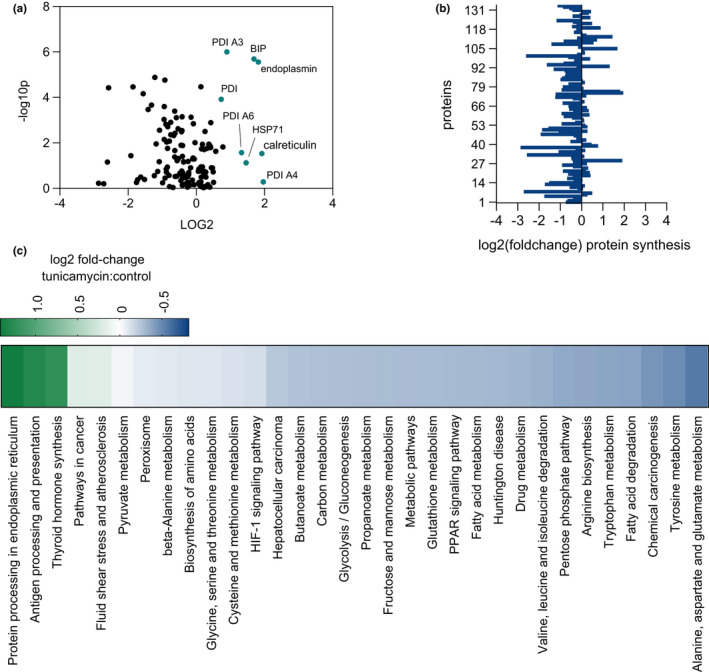
(a) Volcano plot of all proteins for which fractional synthesis rates were measured (*n* = 136) in 80‐week‐old mice. Points expressed as log2 fold‐change tunicamycin treated/control on *x*‐axis and ‐ log10(*p* value), obtained from 2‐tailed *t*‐test (*p* ≤ 0.05), on *y*‐axis. Significantly increased ER stress proteins are highlighted in blue (*p* < 0.05 as determined by 2‐tailed *t*‐test). (b) log2 fold‐change of individual protein translation rates of tunicamycin treated/control. (c) KEGG‐pathway analysis for fractional synthesis rates of proteins from 80‐week‐old mice tunicamycin treated/control. The heat map color code is shown. *n* = at least 5 proteins per pathway

**FIGURE 3:**
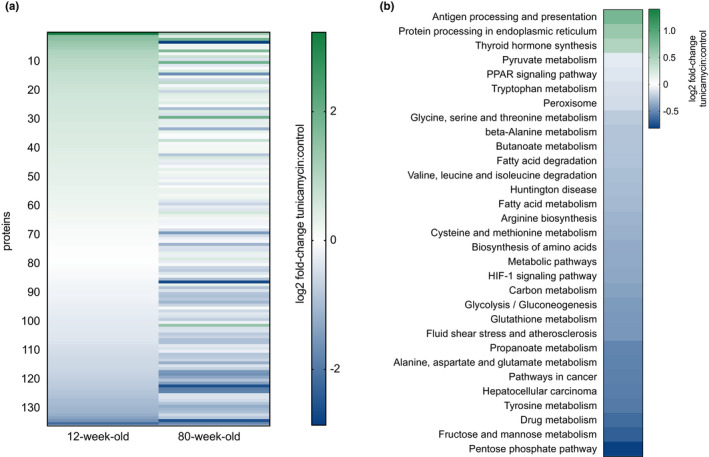
Effects of age on global protein synthesis rates response to tunicamycin treatment for paired proteins in young and aged mice. (a) Proteome‐wide tunicamycin‐induced changes in individual protein translation rates (*n* = 136 proteins). Values expressed as log2 fold‐change of tunicamycin treated/control. 12‐week‐old mice values expressed on the left, and protein matched 80‐week‐old values expressed on the right. (b) KEGG‐pathway analysis of the log2 fold‐change of 80‐week‐old mice treated with tunicamycin compared with 12‐week‐old mice treated with tunicamycin (1 = no change). *n* = at least 5 proteins per pathway

In contrast, aged mice exhibited a strikingly different response to chronic ER stress compared with their younger counterparts (Figure 2a–b). Aged mice experienced broad inhibition of protein translation at 4 days of tunicamycin administration, with most ontologies showing suppression of protein synthesis. Proteins in the ontology *protein processing in the ER* remained more highly upregulated than in the young mice, with a 2.6‐fold increase in synthesis rates compared with tunicamycin challenged young mice. Ontologies pertaining to lipid metabolism, including *PPAR signaling*, *fatty acid metabolism*, and *fatty acid degradation*, were more suppressed in the aged mice as compared with young mice challenged with tunicamycin (Figure 2b). Overall, when challenged with tunicamycin, aged mice showed much lower rates of translation across most ontologies than observed in control age‐matched animals (Figure 3). In contrast, those ontologies that were upregulated remained as highly increased in translation rate as the younger mice. When compared directly, under ER stress conditions, most ontologies in aged mice were significantly suppressed in their synthesis compared with young mice (Figure 3), with the exception of higher rates of synthesis of the ontology *protein processing in the ER*. The proteins included in each of these ontologies are shown (Table S2).

### 2.3 BiP synthesis is higher with aging under ER stress conditions

**FIGURE 4:**
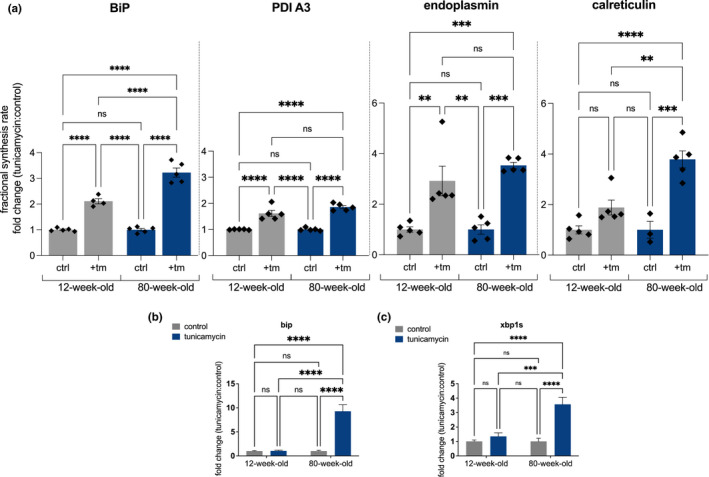
(a) Ratio of changes in fractional synthesis rate in 12‐week‐old and 80‐week‐old mice treated with tunicamycin of ER proteins including BiP, protein disulfide‐isomerase A3, endoplasmin, and calreticulin (*n* = 5 per group). (b–c) (c) RT‐qPCR for *bip* and *xbp1s* in 12‐week‐old and 80‐week‐old mice (*n* = 5). Statistical analysis completed in GraphPad using 2‐way ANOVA. ns, no significance, * = <0.0332, ** = <0.0021, *** = <0.0002, **** = <0.0001

**FIGURE 5:**
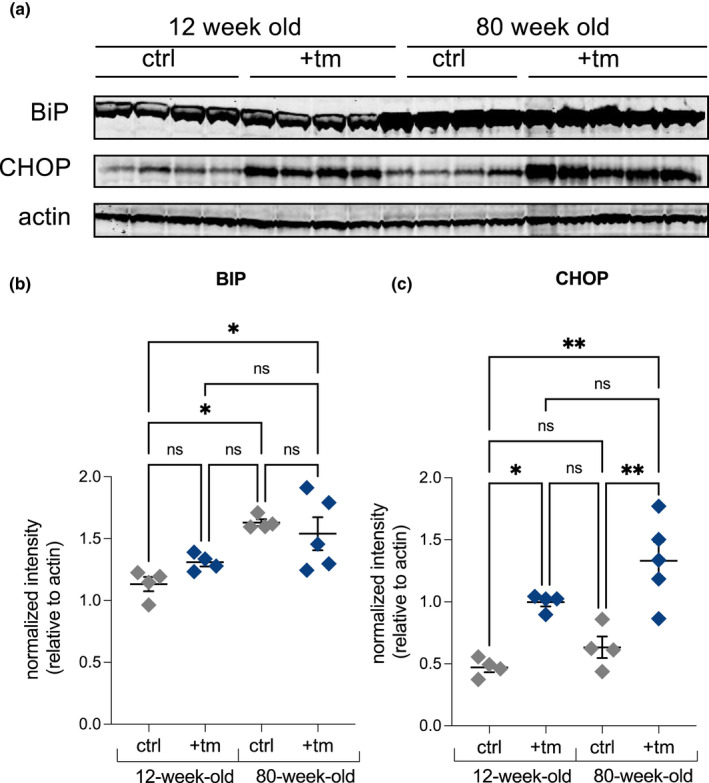
(a) Western blot analysis of BiP and CHOP with actin loading control in 12‐week‐old and 80‐week‐old mice. (b–c) Quantification of Western blots for BiP and CHOP normalized to actin using ImageJ to quantify blot intensity. Statistical analysis completed in GraphPad using 2‐way ANOVA. ns, no significance, * = <0.0332, ** = <0.0021, *** = <0.0002, **** = <0.0001

BiP, a key chaperone involved in the UPR^ER^, was much more highly increased in synthesis rate in aged mice challenged with tunicamycin compared with young mice (Figure 4). BiP synthesis increased by ~2‐fold in young mice under ER stress but by more than 3‐fold in aged mice under ER stress conditions, with a significantly greater increase in translation rate of BiP in aged tunicamycin challenged compared with young (*p* < 0.001). To compare rates of translation to mRNA levels, *bip* mRNA was measured via RT‐qPCR. *bip* mRNA in the liver showed no significant difference in young mice challenged with tunicamycin, however, was 9.2‐fold higher in aged mice challenged with tunicamycin as compared with controls (Figure 4b). *xbp1s* mRNA, a spliced version of *xbp1* indicative of initiation of the UPR^ER^ (Lee et al., 2002), was also measured. Young mice showed no significant differences when challenged with tunicamycin whereas aged mice showed a 3.5‐fold increase (Figure 4c). Abundance of BiP and CHOP proteins was measured via Western blot (Figure 5a). No differences between young and aged were seen in BiP after challenge with tunicamycin; however, aged mice had baseline more BiP than younger mice (Figure 5b). Both young and aged mice had higher levels of CHOP abundance after tunicamycin treatment, with aged mice displaying a higher increase in CHOP abundance (Figure 5c).

### 2.4 De novo lipogenesis is suppressed by both induction of the UPR and age

**FIGURE 6:**
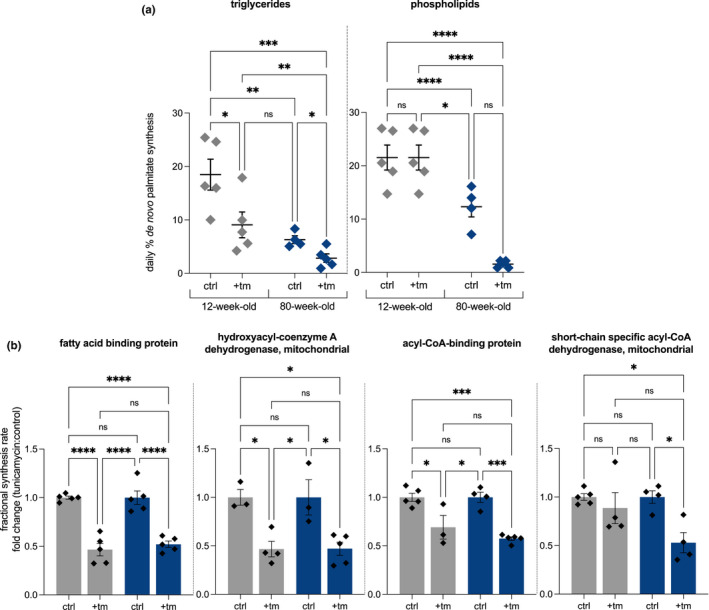
(a) De novo lipogenesis (DNL) fractional contribution to palmitate incorporated into triglycerides in 12‐week‐old and 80‐week‐old control and tunicamycin treated mice (*n* = 5 per group). (b) Fractional replacement rates of proteins involved in lipid synthesis in response to tunicamycin for 12‐week‐old control and and 80‐week‐old tunicamycin treated mice (*n* = 5 per group). Statistical analysis completed in GraphPad using 2‐way ANOVA. ns, no significance, * = <0.0332, ** = <0.0021, *** = <0.0002, **** = <0.0001

The contribution from DNL to liver lipids during the treatment period was measured. Rates of DNL were measured for both palmitate incorporated into hepatic phospholipid and triglyceride fractions. Young mice experiencing chronic ER stress showed a significant reduction in de novo palmitate incorporation into triglycerides but there was no significant change in DNL contribution to phospholipids. In contrast, aged mice experiencing chronic ER stress displayed a significant reduction in de novo palmitate incorporation into both triglyceride and phospholipid fractions (Figure 6a). Fractional synthesis rates of proteins involved in lipid metabolism demonstrated reduction in fatty acid‐binding protein, hydroacyl‐coenzyme A dehydrogenase mitochondrial, acyl‐CoA‐binding protein, and short‐chain specific acyl‐CoA dehydrogenase mitochondrial in aged mice treated with tunicamycin (Figure 6b). Synthesis rates of fatty acid‐binding protein, hydroacyl‐coenzyme A dehydrogenase mitochondrial, and acyl‐CoA‐binding protein were also decreased in young mice treated with tunicamycin (Figure 6b).

## 3 DISCUSSION

Several observations are of interest related to metabolic fluxes induced by chronic ER stress.

### 3.1 Chronic UPR signature in young mice

Young mice challenged with chronic ER stress for 4 days demonstrated increased translation of proteins involved in *protein processing in the ER*, however, showed modest changes in other ontologies. We have previously shown that acute tunicamycin administration in mice markedly reduces protein synthesis across the global proteome in the liver for the first 72 h (Ward et al., 2022). The data here suggest that by Day 4 of repeated tunicamycin treatment, these mice may be recovering from the induced ER stress. In contrast, with chronic ER stress synthesis rates of key UPR^ER^ regulators such as chaperones and BiP remained upregulated. The increase of chaperones and BiP synthesis with suppressed synthesis of proteins involved in lipid metabolism indicates a still partially active UPR^ER^, but with no reduction in most other ontologies. Interestingly, we found significantly increased BiP synthesis rates despite no change in *bip* mRNA levels after UPR^ER^ induction in young mice. This again demonstrates that directly measuring protein synthesis rates may be more sensitive to changes in translational activity than measurement of mRNA levels (Beysen et al., 2019).

### 3.2 Aging exaggerates the UPR metabolic flux signature

Aged mice experiencing chronic ER stress showed a more exaggerated UPR^ER^ signature compared with their young counterparts. Aged mice had suppressed rates of translation across the proteome but sustained the same increase in translation of proteins involved in *protein processing in the ER*. This ontology (Table S2) encompasses many known UPR^ER^ proteins such as BiP and protein disulfide isomerases seen in our data set. BiP synthesis was indeed significantly increased in aged mice challenged with tunicamycin as compared with young mice, which was consistent with *bip* mRNA levels. Abundance of BiP protein by Western blot appeared to be the same when measured in both young and aged mice challenged with tunicamycin, in contrast to higher message levels and increased synthesis rates in aged mice. This may be indicative of more rapid clearance of BiP in aged mice, compensating for higher synthesis rate, or may reflect less sensitivity of the protein concentration measurements. Aged mice also had higher baseline BiP than young mice, indicating that they may have higher baseline ER stress before events of ER stress induction, in this case, tunicamycin treatment. These data highlight the complex translational control mechanisms used to restore protein homeostasis. Overall, we saw suppression of protein synthesis rates for most ontologies in aged mice under ER stress conditions but not in young mice at Day 4 of tunicamycin administration, exemplifying an exaggerated UPR^ER^ in the aged animals. A decrease in glutathione metabolism in aged mice under ER stress conditions compared with young mice was seen as well, which may predispose to altered redox homeostasis (Circu & Aw, 2012).

### 3.3 Dysregulation of lipid metabolism

Ontologies involved in lipid metabolism, such as *fatty acid degradation* and *PPAR signaling*, were more suppressed in aged mice challenged with tunicamycin, which was consistent with our data showing de novo fatty acid synthesis rates were more broadly reduced with aging and ER stress. Aged mice showed a more striking decline in DNL as compared with young mice, specifically in the phospholipid fraction. Phospholipids comprise membranes, so reduced de novo synthesis their ability to expand their ER membranes under states of ER stress (Sriburi et al., 2004). In contrast, young mice exhibited decline in DNL contribution to palmitate incorporated into triglycerides, but not phospholipids. We have previously shown that with acute ER stress, palmitate synthesis and incorporation into phospholipids decline starting 48 h after tunicamycin treatment and remains significantly decreased at 72 h post treatment (Ward et al., 2022). In this chronic ER stress model after 4 days we see no significant decline in DNL of phospholipid fractions, which suggest young mice are better able to resume synthesis of palmitate and subsequent incorporation into phospholipids after 4 days of repeated ER stress. Other groups have seen a similar lower DNL phenotype in vivo as well (DeZwaan‐McCabe et al., 2017; Herrema et al., 2016), indicative of a systematic difference in lipogenic response to UPR^ER^ induction in vivo as compared with isolated cellular models (Lee et al., 2012), which typically show increased rates of lipogenesis under ER stress. These differences can be reconciled by the ability of a whole organism to draw from other tissues for lipid sources in vivo. We have previously shown that fatty acids are taken up by the liver and utilized from other tissues, such as the adipose tissue, under ER stress conditions (Ward et al., 2022). In yeast, ER membrane expansion has been seen with initiation of the UPR; so, it might be speculated that this decrease in phospholipid synthesis in aged mice could hinder their ability to recover from ER stress (Schuck et al., 2009).

We, among other groups, have demonstrated that tunicamycin treatment leads to anorexia in young mice (DeZwaan‐McCabe et al., 2017; Ward et al., 2022). In this study, we measured both food intake and changes in weight as mice were treated with tunicamycin (Figure S2). Although weights trended down in aged mice treated with tunicamycin, the changes in weight compared with control were not statistically significant. We speculate the stress of daily injections may have led to perturbations in food intake. Aged mice also weigh more on average and have greater weight variability within cohorts, thus leading to a range in weight trends. Young mice, however, displayed a significant decrease in weight by the 96 h time point. We previously discovered that when the UPR^ER^ is initiated, lipids are mobilized from the adipose tissue to the liver (Ward et al., 2022).

In addition to the accumulation of misfolded proteins, dietary changes such as high intake of fatty acids can perturb proteostasis and thereby initiate the UPR^ER^ (Fu et al., 2012; Salvadó et al., 2015; Wang & Kaufman, 2016). Lipid bilayer stress is thought to act though IRE1ɑ sensors, initiating downstream effects of this arm of the UPR^ER^, recently demonstrated in yeast (Ho et al., 2020). We demonstrated that the decline in de novo lipogenesis seen under ER stress conditions is exacerbated with age; however, it is unknown that how diet in aged animals affects this metabolic response to ER stress. We might expect that activation of the UPR^ER^ through diet in an aged model could exacerbate the decline in de novo lipogenesis that we observed in aged mice under ER stress. The decline of de novo lipogenesis with age may provide some benefit to handling high‐fat diet‐induced ER stress; however, we hypothesize that since de novo lipogenesis is decreased under ER stress conditions and lipids incorporated into ER stress membranes have been shown to be adipose tissue derived (Ward et al., 2022), this decline in de novo lipogenesis is likely not advantageous for aged animals.

**FIGURE 7:**
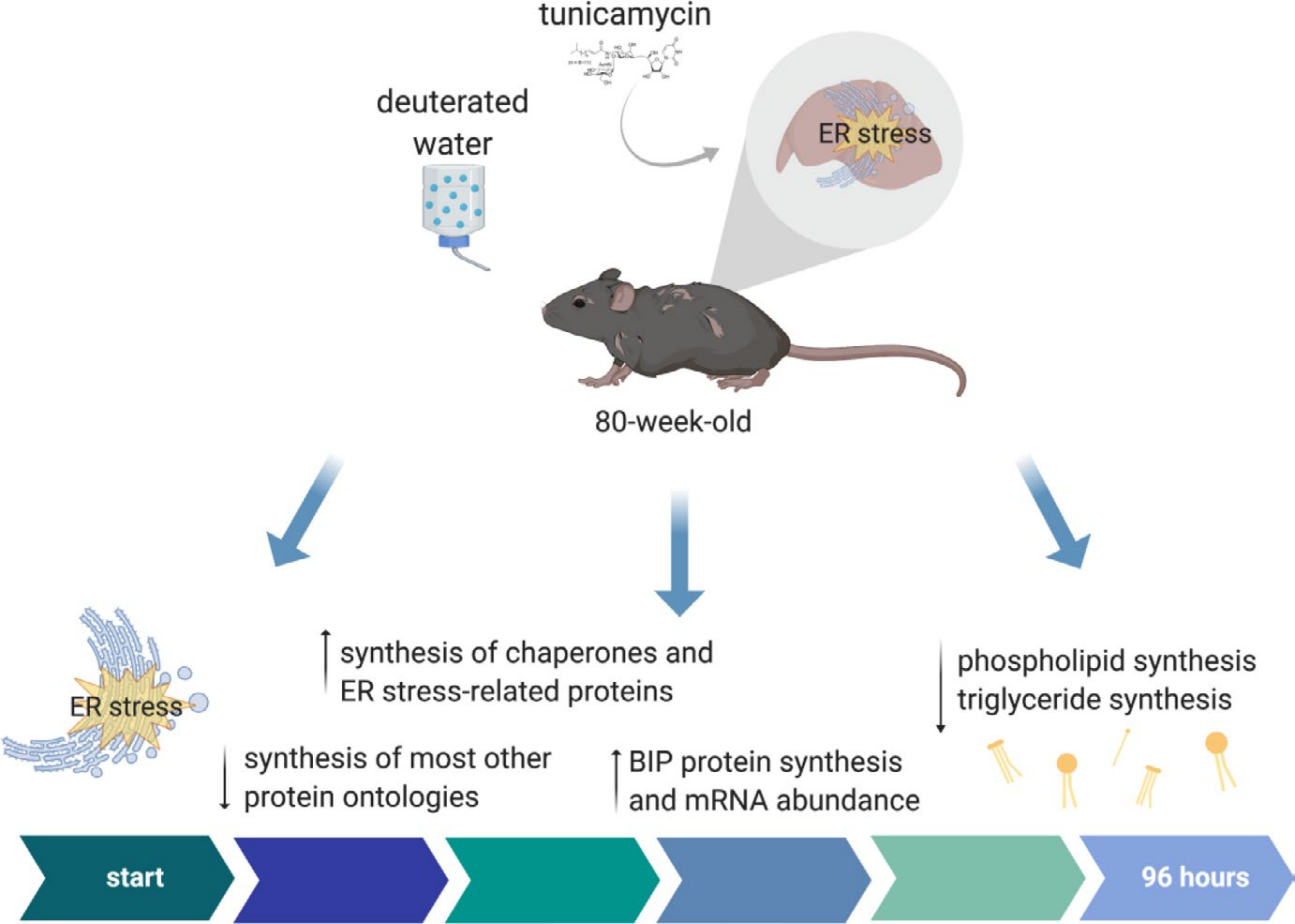
Summary figure. Exaggerated UPR signature in aged animals, with higher translation of ER stress‐related proteins, lower translation of all other proteins, lower rates of de novo lipogenesis, and BiP protein synthesis and mRNA abundance higher in aged animals with tunicamycin‐induced ER stress

We conclude that aging leads to an exaggerated chronic ER stress metabolic signature, including ongoing suppression of synthesis in most protein ontologies, with the exception of *protein processing in the ER*, which remains upregulated (Figure 7). These data in combination with higher BiP synthesis and dysregulated lipid metabolism in aged mice that are challenged with tunicamycin indicate that aged mice are not efficient at handling and recovering from ER stress: they sustained high levels of BiP synthesis throughout the 4‐day treatment period, whereas young mice showed less increase in BiP synthesis and no significant upregulation of *bip* mRNA; they exhibited prolonged suppression of proteome‐wide protein synthesis, whereas young mice showed no general suppression at Day 4 of tunicamycin; and they exhibited much greater suppression of de novo synthesis of liver lipids, particularly phospholipids, which may be required for membrane expansion. Our data therefore suggest that aging leads to impaired efficiency of the UPR^ER^, leading to a prolonged and exaggerated UPR^ER^ metabolic flux signature. This impairment in the UPR^ER^ with age may be a contributing factor to diseases that manifest with age. In the liver, specifically, a less capable UPR^ER^ with aging may contribute to metabolic diseases including non‐alcoholic fatty liver disease.

## 4 EXPERIMENTAL PROCEDURES

### 4.1 Animals

C57BL/6J male mice acquired from The Jackson Laboratory were used for this study. Mice were aged to either 12 or 80 weeks. All mice were housed according to the Animal Care and Use Committee (ACUC) standards in the animal facility at UC Berkeley. Mice were fed a standard chow diet and water ad libitum.

### 4.2 Deuterated water labeling and tunicamycin treatment in mice

Mice were labeled with deuterated water (heavy water, ^2^H_2_O) beginning at time point 0 (t^0^) through the end of the experiment. Proteins synthesized after t^0^ will incorporate deuterium‐labeled amino acids, thus enabling the measurement of proteins synthesized during the period of exposure to heavy water. Deuterium is rapidly incorporated throughout the body of an organism after treatment, bringing the deuterium enrichment in body water up to 5%. Deuterium enrichment is maintained through the intake of 8% ^2^H_2_O given as drinking water, thus making it an optimal labeling approach for in vivo experimental study. Mice are injected intraperitoneally (IP) with 100% ^2^H_2_O containing either tunicamycin dissolved in DMSO, or DMSO control. Mice were treated at 1 mg/kg tunicamycin one per day, or no drug control, and tissues were harvested 96 hours after injections (*n* = 5 mice per group).

### 4.3 Body water enrichment analysis

Mouse livers were distilled overnight upside down on a bead bath at 85°C to evaporate out body water. Deuterium present in the body water was exchanged into acetone, and deuterium enrichment in the body water was measured via gas chromatography–mass spectrometry (GC‐MS; Busch et al., 2007; Turner et al., 2003).

### 4.4 Tissue preparation for liquid chromatography–mass spectrometry (LC‐MS)

Tissues were flash frozen after harvest and homogenized in homogenization buffer (100 mM PMSF, 500 mM EDTA, EDTA‐free Protease Inhibitor Cocktail (Roche, catalog number 11836170001), PBS) using a 5 mm stainless steel bead at 30 hertz for 45 s in a TissueLyser II (Qiagen). Samples were then centrifuged at 10,000 rcf for 10 min at 4°C. The supernatant was saved, and protein was quantified using a Pierce BCA protein assay kit (ThermoFisher, catalog number 23225). One hundered microgram of protein was used per sample. Twenty‐five microlitre of 100 mM ammonium bicarbonate solution, 25 µl TFE, and 2.3 µl of 200 mM DTT were added to each sample and incubated at 60°C for 1 h. Ten microlitre 200 mM iodoacetamide was then added to each sample and allowed to incubate at room temperature in the dark for 1 h. Two microlitre of 200 mM DTT was added, and samples were incubated for 20 min in the dark. Each sample was then diluted with 300 µL H_2_O and 100 µL 100 mM ammonium bicarbonate solution. Trypsin was added at a ratio of 1:50 trypsin to protein (trypsin from porcine pancreas, Sigma Aldrich, catalog number T6567). Samples were incubated at 37°C overnight. The next day, 2 µL of formic acid was added. Samples were centrifuged at 10,000 rcf for 10 min, collecting the supernatant. Supernatant was dried in a speedvac and re‐suspended in 50 µL of 0.1% formic acid/3% acetonitrile/96.9% LC‐MS grade water and transferred to LC‐MS vials to be analyzed via LC‐MS.

### 4.5 Liquid chromatography–mass spectrometry analysis

Trypsin‐digested peptides were analyzed on a 6550 quadrupole time of flight (Q‐ToF) mass spectrometer equipped with Chip Cube nano ESI source (Agilent Technologies). High performance liquid chromatography (HPLC) separated the peptides using capillary and nano binary flow. Mobile phases were 95% acetonitrile/0.1% formic acid in LC‐MS grade water. Peptides were eluted at 350 nl/min flow rate with an 18 min LC gradient. Each sample was analyzed once for protein/peptide identification in data‐dependent MS/MS mode and once for peptide isotope analysis in MS mode. Acquired MS/MS spectra were extracted and searched using Spectrum Mill Proteomics Workbench software (Agilent Technologies) and a mouse protein database (www.uniprot.org). Search results were validated with a global false discovery rate of 1%. A filtered list of peptides was collapsed into a nonredundant peptide formula database containing peptide elemental composition, mass, and retention time. This was used to extract mass isotope abundances (M0‐M3) of each peptide from MS‐only acquisition files with MassHunter Qualitative Analysis software (Agilent Technologies). Mass isotopomer distribution analysis (MIDA; Hellerstein & Neese, 1992, 1999; Holmes et al., 2015) was used to calculate peptide elemental composition and curve‐fit parameters for predicting peptide isotope enrichment based on precursor body water enrichment (p) and the number (n) of amino acid C‐H positions per peptide actively incorporating hydrogen (H) and deuterium (D) from body water. Subsequent data handling was performed using python‐based scripts, with input of precursor body water enrichment for each subject, to yield fractional synthesis rate (FSR) data at the protein level. FSR data were filtered to exclude protein measurements with fewer than 2 peptide isotope measurements per protein. Details of FSR calculations and data filtering criteria have been described in detail previously(Holmes et al., 2015; Thompson et al., 2016).

### 4.6 Calculation of fractional replacement (f) and replacement rate constant (k) for individual proteins

Details of calculations were previously described (Holmes et al., 2015). These values were used to generate the ratio of tunicamycin treated to untreated synthesis rates.

### 4.7 Statistical analysis

Data were analyzed using GraphPad Prism software (versions 8.0‐9.0). We used 2‐way ANOVA analysis in Prism GraphPad for fractional synthesis displayed as individual proteins, Western blots, and qPCR. We used 2‐tailed *t*‐tests for analysis of global fractional synthesis rates for heat maps.

### 4.8 Tissue preparation for gas chromatography–mass spectrometry

A chloroform methanol extraction was used to isolate lipids from the liver tissue. These lipids were run on a thin‐layer chromatography (TLC) plate to separate phospholipid and triglyceride fractions. These fractions containing the palmitate were further derivatized for GC‐MS analysis.

### 4.9 Gas chromatography–mass spectrometry analysis

Palmitate isotopic enrichments were measured by GC‐MS (Agilent models 6890 and 5973; Agilent, Inc.) using helium carrier gas, a DB‐225 (DB‐17 for cholesterol and DB‐225 for palmitates) fused silica column (30 M × 0.25 mm ID × 0.25 µm), electron ionization mode, and monitoring m/z 385, 386, and 387 for palmitates, and 368, 369, 370 for cholesterol acetyl derivatives, for M0, M1, and M2, respectively, as previously described(Hellerstein et al., 1991, 1997). Palmitate methyl ester enrichments were determined by GC‐MS using a DB‐17 column (30 M × 0.25 mm ID × 0.25 µm), with helium as carrier gas, electron ionization mode, and monitoring m/z 270, 271, and 272 for M0, M1, and M2. Baseline unenriched standards for both analytes were measured concurrently to correct for abundance sensitivity.

### 4.10 Calculation of de novo lipogenesis

The measurement of newly synthesized fatty acids and total cholesterol formed during ^2^H_2_O labeling period was assessed using a combinatorial model of polymerization biosynthesis, as described previously(Hellerstein et al., 1991, 1996; Neese et al., 1993). Mass isotopomer distribution analysis along with body ^2^H_2_O enrichment, representing the precursor pool enrichment (p), is used to determine the theoretical maximum enrichment of each analyte(Hellerstein et al., 1991; Hellerstein & Neese, 1992, 1999; Neese et al., 1993). Using the measured deuterium enrichments, fractional and absolute contributions from DNL are then calculated. The value for f DNL represents the fraction of total triglyceride or phospholipid palmitate in the depot derived from DNL during the labeling period, and absolute DNL represents grams of palmitate synthesized by the DNL pathway.

### 4.11 Western blot

Starting with frozen tissue, tissue was homogenized in homogenization buffer (100 mM PMSF, 500 mM EDTA, EDTA‐free Protease Inhibitor Cocktail (Roche, catalog number 11836170001), PBS) using a 5 mm stainless steel bead at 30 hertz for 45 s in a TissueLyser II (Qiagen). Samples were then centrifuged at 10,000 rcf for 10 min at 4°C. The supernatant was saved, and protein was quantified using a Pierce BCA protein assay kit (ThermoFisher, catalog number 23225). Thirty microgram of protein was used per sample. 2X Laemmli sample buffer was added (Sigma, catalog number S3401) at a 1:1 ratio. Samples were brought to the same volume with 1% SDS, vortexed briefly, and heated in a heating block for 10 min at 95°C. Samples were tip sonicated for 10 s and then centrifuged for 5 min at 15,000 g. 4%–12% gradient poly‐acrylamide gels were used with MES buffer, and gels were run at 120 V until loading dye line passed through gel. iBlot2 was used to transfer the gel to a PVDF membrane. Membranes were washed 3 times with PBST and then blocked with 5% milk for 1 h. Membranes were then washed again with PBST 3 times. BiP (Cell Signaling Technology, catalog number 3183S) and actin (Santa Cruz technology, catalog number 47778) antibodies were diluted in 5% BSA and rotated at 4°C overnight. Membranes were then washed 3 times with PBST, and LiCor secondary antibodies diluted in 5% BSA were added and rotated for 2 h at room temperature. Membranes were washed 3 times with PBST and then imaged using a LiCor imaging system.

### 4.12 Quantitative reverse transcription PCR (RT‐qPCR)

RNA was isolated using standard Trizol protocol, and RNA concentrations were obtained using a Nanodrop. After normalizing concentrations, cDNA was synthesized using 2 µg RNA with RevertAid RT Kit (Thermofisher, catalog number K1691). Maxima SYBR Green/ROX qPCR Master Mix (ThermoFisher, catalog number K0221) was used for RT‐qPCR. Actin was used to normalize. Oligonucleotide sequences used were as follows:

bip F: CGAGGAGGAGGACAAGAAGG.

bip R: CACCTTGAACGGCAAGAACT.

xbp1s forward: TGCTGAGTCCGCAGCAGGTG.

xbp1s reverse: GCTGGCAGGCTCTGGGGAAG.

actin forward: CGAATCATGAGCATTGTAGAC.

actin reverse: GTAATTCTTATCTCCAGCCAG.

### 4.13 KEGG‐pathway analysis

Protein fractional synthesis rates were weighted by the peptide count and averaged according to their KEGG‐pathway involvements. We used the Uniprot.ws package in R from Bioconductor to find mappings between UniProt accession numbers and their corresponding KEGG IDs for each protein. Tables were generated for the entire known proteome for mouse. We then used the Bio.KEGG module of Biopython in Python to access to the REST API of the KEGG database to get a list of pathways to which each protein belongs. A set of all the pathways relevant to the experiment was generated, and each protein and its corresponding fold‐change value were assigned to each pathway. KEGG‐pathways with no less than five proteins were used for representation of the data.

## CONFLICT OF INTEREST

The authors deny any conflict of interest.

## AUTHOR CONTRIBUTIONS

CPW, AD, and MKH conceived and designed experiments. CPW, MD, HKG, LP, SY, and JH performed experiments. CPW and MKK analyzed and interpreted the data. HP assisted with tunicamycin treatment in mice. HM and EN assisted with LC‐MS and GC‐MS sample processing and data analysis. NZ performed KEGG‐pathway analysis. CPW, AEF, and MKH contributed in writing the manuscript.

### OPEN RESEARCH BADGES

This article has earned an Open Data Badge for making publicly available the digitally‐shareable data necessary to reproduce the reported results. The data is available at https://www.biorxiv.org/content/10.1101/2021.04.14.439896v1.

## Supporting information

Data S1

Data S2

Supplementary Material

## Data Availability

The data that support the findings of this study are openly available as [Link], [Link] to our publication.
