# Supplementary material for: Aging alters the metabolic flux signature of the ER‐unfolded protein response in vivo in mice
Source: Aging Cell. 2022 Feb 16;21(3):e13558. doi: 10.1111/acel.13558 (PMC8920450; doi:10.1111/acel.13558)
Supplement: Supplementary file 3 — Supplementary Material [file ACEL-21-e13558-s001.docx]

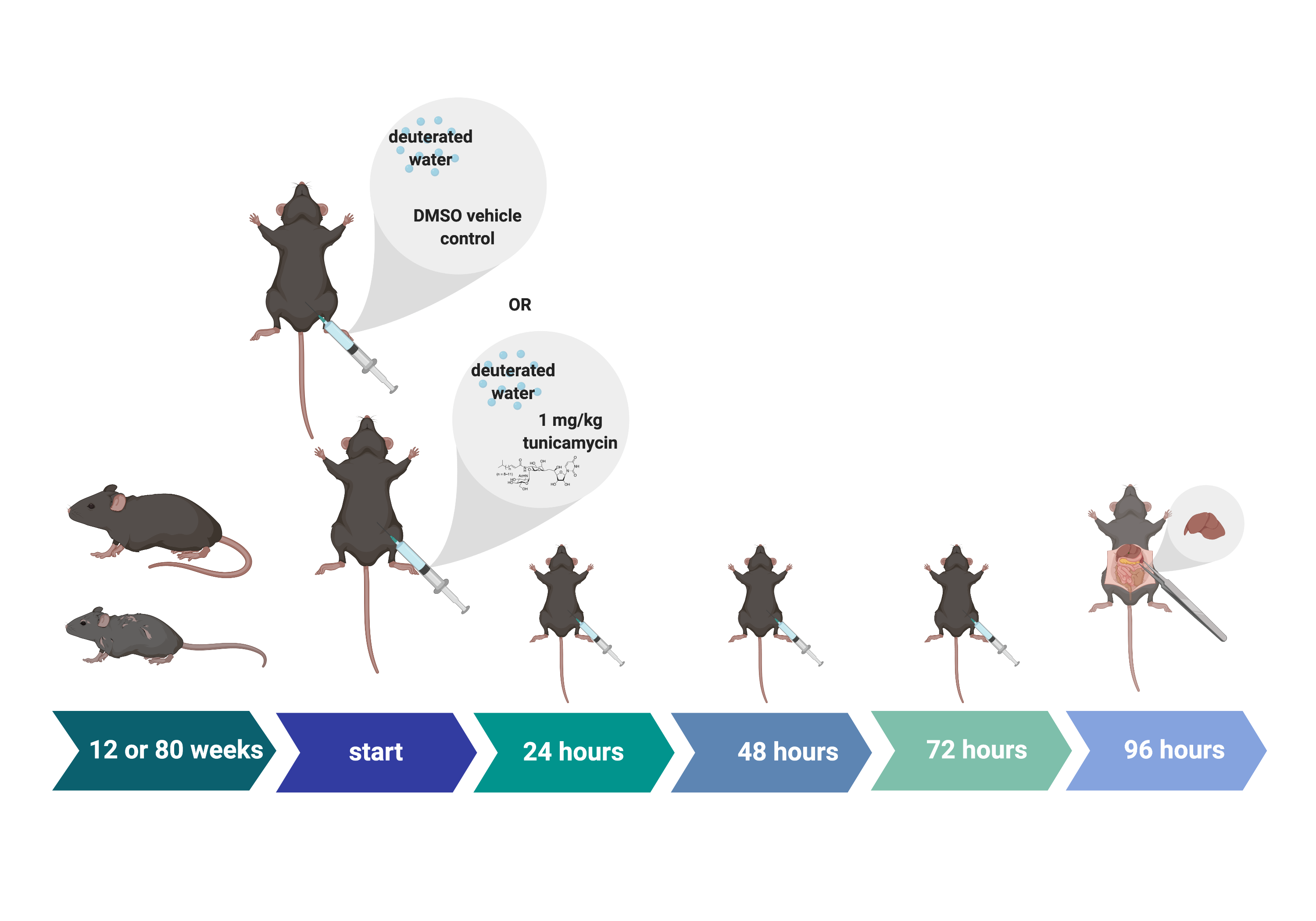


Supplementary Figure 1. Study design: 12-week-old or 80-week-old mice (n=5 per group) were treated with 1 mg/kg tunicamycin or vehicle control, DMSO, once per day for 3 days. Mice were injected with 35uL/g deuterated water on day 1. Mice were sacrificed and livers were taken on day 4.

Supplementary Figure 2: (a) Average food consumed per mouse per day. (b) Average weight of each mouse at each timepoint. ns= no significance, * = <0.05.

Supplementary Table 1: Individual protein synthesis rate ratios in young and aged mice treated with tunicamycin compared to controls. Ratio of above 1 indicates a higher synthesis rate with age and/or tunicamycin treatment. Ratio of below 1 indicates a lower synthesis rate with age and/or tunicamycin treatment.

| **Protein** | **ratio aged tm/control** | **ratio young tm/control** | **ratio aged tm/young tm** |
| --- | --- | --- | --- |
| 10 kDa heat shock protein, mitochondrial | 1.21 | 1.11 | 1.10 |
| 2-iminobutanoate/2-iminopropanoate deaminase | 0.83 | 1.00 | 0.83 |
| 3-hydroxyacyl-CoA dehydrogenase type-2 | 0.35 | 1.06 | 0.33 |
| 3-ketoacyl-CoA thiolase A, peroxisomal | 1.41 | 1.00 | 1.41 |
| 3-ketoacyl-CoA thiolase B, peroxisomal | 0.76 | 0.79 | 0.95 |
| 3-ketoacyl-CoA thiolase, mitochondrial | 0.76 | 0.89 | 0.85 |
| 40S ribosomal protein S4, X isoform | 1.42 | 1.31 | 1.08 |
| 60 kDa heat shock protein, mitochondrial | 1.10 | 1.19 | 0.92 |
| 60S ribosomal protein L29 | 0.90 | 1.08 | 0.83 |
| 60S ribosomal protein L6 | 1.72 | 1.38 | 1.24 |
| Actin, alpha skeletal muscle | 1.17 | 1.19 | 0.98 |
| Actin, cytoplasmic 2 | 1.11 | 1.11 | 1.00 |
| Acyl-CoA synthetase family member 2, mitochondrial | 0.43 | 0.48 | 0.88 |
| Acyl-CoA-binding protein | 0.57 | 0.69 | 0.83 |
| Acyl-coenzyme A synthetase ACSM1, mitochondrial | 1.05 | 1.50 | 0.70 |
| Adenosylhomocysteinase | 1.10 | 1.37 | 0.80 |
| Alanine--glyoxylate aminotransferase 2, mitochondrial | 0.30 | 1.69 | 0.18 |
| Alcohol dehydrogenase 1 | 0.43 | 0.67 | 0.64 |
| Alcohol dehydrogenase class-3 | 0.58 | 0.78 | 0.74 |
| Aldehyde dehydrogenase family 8 member A1 | 0.96 | 1.39 | 0.69 |
| Aldehyde dehydrogenase, cytosolic 1 | 0.64 | 1.17 | 0.55 |
| Aldehyde dehydrogenase, mitochondrial | 0.73 | 0.79 | 0.92 |
| Alpha-1-antitrypsin 1-3 | 0.84 | 1.41 | 0.60 |
| Alpha-enolase | 0.77 | 0.94 | 0.81 |
| Arginase-1 | 0.77 | 1.13 | 0.68 |
| Argininosuccinate synthase | 1.11 | 1.34 | 0.83 |
| Aspartate aminotransferase, mitochondrial | 0.80 | 1.30 | 0.62 |
| ATP synthase subunit alpha, mitochondrial | 0.73 | 1.02 | 0.72 |
| ATP synthase subunit beta, mitochondrial | 0.69 | 1.06 | 0.66 |
| Betaine--homocysteine S-methyltransferase 1 | 0.69 | 0.50 | 1.38 |
| Bifunctional epoxide hydrolase 2 | 0.78 | 1.24 | 0.63 |
| Calreticulin | 3.79 | 1.88 | 2.01 |
| Carbamoyl-phosphate synthase [ammonia], mitochondrial | 0.64 | 0.97 | 0.66 |
| Carbonic anhydrase 2 | 0.15 | 0.91 | 0.17 |
| Carbonic anhydrase 3 | 0.17 | 0.33 | 0.51 |
| Carboxylesterase 1D | 0.26 | 0.57 | 0.46 |
| Carboxylesterase 1F | 0.34 | 0.65 | 0.52 |
| Carboxylesterase 3A | 0.38 | 0.60 | 0.63 |
| Carboxylesterase 3B | 0.56 | 0.39 | 1.45 |
| Catalase | 0.74 | 0.72 | 1.03 |
| Clathrin heavy chain 1 | 0.86 | 1.04 | 0.82 |
| Cystathionine gamma-lyase | 1.13 | 2.34 | 0.48 |
| Cysteine sulfinic acid decarboxylase | 0.16 | 0.59 | 0.28 |
| Cytochrome c oxidase subunit 6A1, mitochondrial | 1.22 | 1.00 | 1.21 |
| Cytochrome P450 2D10 | 0.94 | 1.85 | 0.51 |
| Cytosolic 10-formyltetrahydrofolate dehydrogenase | 0.53 | 0.93 | 0.57 |
| D-dopachrome decarboxylase | 1.19 | 1.20 | 0.99 |
| Delta-1-pyrroline-5-carboxylate dehydrogenase, mitochondrial | 0.65 | 0.68 | 0.96 |
| Electron transfer flavoprotein subunit alpha, mitochondrial | 0.97 | 1.08 | 0.90 |
| Electron transfer flavoprotein subunit beta | 0.84 | 1.16 | 0.73 |
| Elongation factor 1-alpha 1 | 1.29 | 1.41 | 0.91 |
| Elongation factor 2 | 0.86 | 0.70 | 1.22 |
| Endoplasmic reticulum chaperone BiP | 3.22 | 2.11 | 1.53 |
| Endoplasmin | 3.53 | 2.92 | 1.21 |
| Estradiol 17 beta-dehydrogenase 5 | 0.32 | 0.64 | 0.50 |
| Fatty acid synthase | 0.93 | 1.61 | 0.58 |
| Fatty acid-binding protein, liver | 0.52 | 0.47 | 1.12 |
| Ferritin light chain 1 | 1.10 | 1.25 | 0.88 |
| Formimidoyltransferase-cyclodeaminase | 1.39 | 1.75 | 0.79 |
| Fructose-1,6-bisphosphatase 1 | 1.33 | 1.50 | 0.88 |
| Fructose-bisphosphate aldolase B | 0.84 | 1.31 | 0.64 |
| Fumarylacetoacetase | 0.75 | 1.02 | 0.74 |
| Glutamate dehydrogenase 1, mitochondrial | 0.55 | 0.61 | 0.89 |
| Glutathione peroxidase 1 | 0.65 | 0.78 | 0.83 |
| Glutathione S-transferase A1 | 0.52 | 0.76 | 0.69 |
| Glutathione S-transferase A3 | 0.47 | 0.87 | 0.54 |
| Glutathione S-transferase Mu 1 | 1.17 | 2.50 | 0.47 |
| Glutathione S-transferase Mu 3 | 1.34 | 2.95 | 0.46 |
| Glutathione S-transferase P 1 | 0.52 | 0.77 | 0.68 |
| Glyceraldehyde-3-phosphate dehydrogenase | 0.85 | 1.26 | 0.67 |
| Glycine N-methyltransferase | 1.26 | 1.53 | 0.83 |
| Glycogen phosphorylase, liver form | 0.56 | 0.70 | 0.80 |
| Glyoxylate reductase/hydroxypyruvate reductase | 1.11 | 1.33 | 0.83 |
| Heat shock cognate 71 kDa protein | 2.75 | 0.80 | 3.44 |
| Heat shock protein HSP 90-beta | 0.75 | 1.47 | 0.51 |
| Hemoglobin subunit alpha | 0.60 | 0.81 | 0.73 |
| Hemoglobin subunit beta-1 | 0.63 | 0.85 | 0.74 |
| Homogentisate 1,2-dioxygenase | 0.50 | 1.50 | 0.34 |
| Hydroxyacyl-coenzyme A dehydrogenase, mitochondrial | 0.47 | 0.47 | 1.01 |
| Hydroxymethylglutaryl-CoA lyase, mitochondrial | 1.07 | 0.99 | 1.08 |
| Hydroxymethylglutaryl-CoA synthase, mitochondrial | 0.96 | 1.28 | 0.75 |
| Isochorismatase domain-containing protein 2A | 0.77 | 1.88 | 0.41 |
| Isocitrate dehydrogenase [NADP] cytoplasmic | 1.07 | 0.84 | 1.27 |
| L-lactate dehydrogenase A chain | 0.86 | 1.00 | 0.86 |
| Major urinary protein 1 | 0.55 | 0.89 | 0.61 |
| Major urinary protein 17 | 0.67 | 0.84 | 0.80 |
| Major urinary protein 2 | 0.46 | 1.03 | 0.44 |
| Malate dehydrogenase, cytoplasmic | 1.05 | 1.39 | 0.76 |
| Malate dehydrogenase, mitochondrial | 1.32 | 1.59 | 0.83 |
| Maleylacetoacetate isomerase | 1.20 | 1.21 | 0.99 |
| Medium-chain specific acyl-CoA dehydrogenase, mitochondrial | 0.44 | 1.41 | 0.31 |
| Methanethiol oxidase | 0.75 | 0.55 | 1.37 |
| Methylmalonate-semialdehyde dehydrogenase [acylating], mitochondrial | 1.16 | 1.27 | 0.92 |
| Microsomal glutathione S-transferase 1 | 0.80 | 0.84 | 0.96 |
| NADP-dependent malic enzyme | 0.75 | 0.52 | 1.44 |
| Non-specific lipid-transfer protein | 0.58 | 0.88 | 0.65 |
| Nucleoside diphosphate kinase B | 1.48 | 1.11 | 1.33 |
| Ornithine carbamoyltransferase, mitochondrial | 0.75 | 0.67 | 1.11 |
| Peptidyl-prolyl cis-trans isomerase A | 0.89 | 1.10 | 0.81 |
| Peroxiredoxin-1 | 0.78 | 1.17 | 0.67 |
| Peroxiredoxin-6 | 0.81 | 0.74 | 1.10 |
| Peroxisomal acyl-coenzyme A oxidase 1 | 1.34 | 1.54 | 0.87 |
| Phenylalanine-4-hydroxylase | 1.27 | 1.27 | 0.99 |
| Phosphoglucomutase-1 | 0.76 | 1.89 | 0.40 |
| Phosphoglycerate kinase 1 | 1.21 | 1.40 | 0.87 |
| Phosphoglycerate mutase 1 | 0.73 | 0.82 | 0.90 |
| Polycystic kidney disease protein 1-like 1 | 0.30 | 0.22 | 1.35 |
| Pregnancy zone protein | 1.18 | 1.52 | 0.77 |
| Protein disulfide-isomerase | 1.66 | 1.99 | 0.83 |
| Protein disulfide-isomerase A3 | 1.86 | 1.62 | 1.15 |
| Protein disulfide-isomerase A4 | 3.90 | 1.46 | 2.67 |
| Protein disulfide-isomerase A6 | 2.51 | 9.38 | 0.27 |
| Protein/nucleic acid deglycase DJ-1 | 0.94 | 1.38 | 0.68 |
| Pyruvate carboxylase, mitochondrial | 0.62 | 0.66 | 0.94 |
| Regucalcin | 0.28 | 0.55 | 0.51 |
| Retinal dehydrogenase 1 | 0.59 | 0.97 | 0.61 |
| S-adenosylmethionine synthase isoform type-1 | 1.36 | 1.48 | 0.92 |
| S-formylglutathione hydrolase | 1.06 | 2.05 | 0.52 |
| S-methylmethionine--homocysteine S-methyltransferase BHMT2 | 0.54 | 0.50 | 1.09 |
| Sarcosine dehydrogenase, mitochondrial | 1.24 | 0.90 | 1.38 |
| SEC14-like protein 2 | 0.88 | 0.80 | 1.09 |
| Selenium-binding protein 2 | 0.67 | 0.43 | 1.54 |
| Serum albumin | 0.40 | 0.63 | 0.64 |
| Short-chain specific acyl-CoA dehydrogenase, mitochondrial | 0.53 | 0.89 | 0.60 |
| Sorbitol dehydrogenase | 0.56 | 1.32 | 0.42 |
| Succinate--CoA ligase [ADP/GDP-forming] subunit alpha, mitochondrial | 0.14 | 2.61 | 0.05 |
| Superoxide dismutase [Cu-Zn] | 0.72 | 1.07 | 0.67 |
| Transketolase | 0.68 | 1.68 | 0.40 |
| Trifunctional enzyme subunit alpha, mitochondrial | 0.92 | 1.04 | 0.89 |
| Triokinase/FMN cyclase | 0.54 | 0.90 | 0.60 |
| Triosephosphate isomerase | 0.84 | 1.45 | 0.57 |
| Tubulin beta-2B chain | 1.09 | 1.27 | 0.86 |
| Tubulin beta-4B chain | 1.04 | 1.21 | 0.86 |
| Valacyclovir hydrolase | 1.13 | 0.94 | 1.20 |
| Xanthine dehydrogenase/oxidase | 1.12 | 1.68 | 0.67 |
| Xylulose kinase | 0.65 | 1.56 | 0.41 |
